# SPFDet: CLIP Text-Prior-Guided Structure-Enhanced Detector for Fine-Grained Ship Detection in Remote Sensing Images

**DOI:** 10.3390/s26144476

**Published:** 2026-07-14

**Authors:** Junbo Zhang, Yuheng Li

**Affiliations:** 1 School of Data Science and Big Data Technology, Central South University, Changsha 410083, China; 0106230118@csu.edu.cn; 2 School of Cyberspace Security (School of Cryptology), Hainan University, Haikou 570228, China

**Keywords:** ship detection, remote sensing, CLIP text prior, semantic prior, structure-enhanced transformer, vision-language model, object detection

## Abstract

Ship detection in remote sensing images is crucial for maritime surveillance, port management, and national security. However, existing detectors struggle with complex harbor backgrounds, large-scale variations, and fine-grained inter-class similarities among diverse ship categories. In this paper, we propose SPFDet, a CLIP text-prior-guided structure-enhanced detector that systematically addresses these challenges by integrating vision-language semantic priors with channel-selective feature enhancement. First, a Semantic Prior Component (SPC) employs a frozen CLIP text encoder to generate category-level semantic embeddings from textual descriptions of ship types, which are combined with visual scene priors and detail-aware priors to provide hierarchical domain-specific guidance. Second, a Structure-Enhanced Transformer Module (SETM), equipped with a Cross-level Channel Selection Module (CCSM) and Multi-Head Cross-Attention (MHCA), selectively enhances discriminative channel responses associated with hull contours, deck textures, and fine structural patterns across encoder layers. Third, a Fused Category-Dominated Feature (FCDF) generation mechanism integrates CLIP-based semantic priors with multi-scale visual features through category-guided attention, producing category-aware representations for precise classification and localization. We further introduce a Category-Semantic Consistency Loss to enforce alignment between predicted features and CLIP text priors. Extensive experiments on HRSC2016 and ShipRSImageNet benchmarks demonstrate that SPFDet achieves 97.2% and 72.8% mAP respectively, providing consistent improvements over strong detection baselines while maintaining competitive inference speed.

## 1. Introduction

Ship detection in remote sensing images has become a fundamental task in earth observation, serving critical applications including maritime traffic monitoring, illegal fishing surveillance, port logistics management, and national defense [1,2]. With the rapid advancement of satellite imaging technology and the proliferation of high-resolution optical sensors, an unprecedented volume of remote sensing data is now available, demanding automated and accurate ship detection algorithms [3].

Deep learning-based object detection methods have achieved remarkable progress in natural image domains [4,5,6]. However, directly applying these general-purpose detectors to ship detection in remote sensing images introduces several unique challenges. First, remote sensing images exhibit extreme scale variations—ships range from small fishing boats spanning only a few pixels to large aircraft carriers occupying hundreds of pixels, making multi-scale feature representation essential. Second, complex harbor backgrounds containing docks, buildings, and water reflections create substantial visual clutter that degrades detection precision [7]. Third, fine-grained ship categories (e.g., destroyers, cruisers, and frigates) share highly similar visual appearances, requiring the detector to capture subtle discriminative features for accurate classification [8].

Recent advances in remote sensing object detection have explored various directions to address these issues. Two-stage detectors such as Faster R-CNN [4] and Cascade R-CNN [9] provide high accuracy but suffer from slow inference. One-stage detectors including RetinaNet [5] and FCOS [10] achieve better speed-accuracy trade-offs but lack fine-grained discriminative capability. The YOLO family [11,12,13] delivers real-time performance yet often struggles with densely packed ships in harbors. Transformer-based detectors such as DETR [6], Deformable DETR [14], and RT-DETR [15] leverage global attention mechanisms but treat all object categories uniformly, ignoring the domain-specific semantic structure inherent in ship detection scenarios. Moreover, recent works on multi-modal remote sensing detection [16,17] and intelligent visual recognition [18] have demonstrated the importance of incorporating prior knowledge and cross-modal feature fusion for improving detection performance in complex scenes.

A critical observation motivating our work is that ships in remote sensing images possess strong categorical semantic priors. Different ship types exhibit distinctive structural patterns: warships feature elongated hulls with weapon systems, cargo ships have large container areas, and submarines present unique low-profile silhouettes. These categorical patterns provide valuable guidance that existing detectors fail to exploit. Furthermore, ship structures contain regular spatial patterns (e.g., deck layouts, container arrangements) that are reflected in multi-channel feature responses related to contours, textures, and local structural repetitions [19,20], suggesting that channel-selective feature enhancement can complement spatial-domain representations.

Recent advances in Vision-Language Models (VLMs) have demonstrated remarkable capabilities in encoding rich semantic knowledge about visual concepts [21,22]. CLIP [21], trained on massive image-text pairs, learns textual representations that capture fine-grained categorical attributes and structural characteristics while remaining aligned with visual features. Leveraging CLIP text embeddings as category-level priors offers a promising yet largely unexplored avenue for remote sensing detection, as textual descriptions of ship categories can encode discriminative structural knowledge (e.g., “an aircraft carrier with a flat flight deck and island superstructure”) that complements purely visual features. Meanwhile, recent works on advanced target recognition in remote sensing [23,24,25] have further highlighted the importance of incorporating external knowledge and robust feature learning for accurate recognition in complex scenes.

Based on these insights, we propose SPFDet (CLIP text-prior-guided Structure-enhanced Detector), a detection framework that integrates CLIP-based semantic knowledge with structure-aware channel enhancement. Our key contributions are summarized as follows:

- We propose a Semantic Prior Component (SPC) that leverages a frozen CLIP text encoder to generate category-level semantic embeddings from textual descriptions of ship types, combined with visual scene priors and detail-aware priors, to provide hierarchical domain-specific guidance. Unlike conventional detectors that rely solely on data-driven feature extraction, SPC introduces vision-language semantic priors that improve fine-grained ship classification.
- We design a Structure-Enhanced Transformer Module (SETM) that integrates a Cross-level Channel Selection Module (CCSM) and Multi-Head Cross-Attention (MHCA) to selectively enhance discriminative channel responses across multi-scale encoder features. SETM strengthens structure-sensitive representations, enabling the detector to capture subtle differences among ship categories.
- We introduce a Fused Category-Dominated Feature (FCDF) generation mechanism that fuses semantic priors with multi-scale visual features through category-guided attention, along with a Category-Semantic Consistency Loss that enforces alignment between predicted features and semantic priors during training.
- Extensive experiments on HRSC2016 [26] and ShipRSImageNet [8] demonstrate that SPFDet achieves competitive state-of-the-art performance. Ablation studies and visualization analyses validate the effectiveness of each proposed component.

The remainder of this paper is organized as follows. Section 2 reviews related work. Section 3 details the proposed SPFDet framework. Section 4 presents experimental results. Section 5 provides discussion, and Section 6 concludes the paper.

## 2. Related Work

### 2.1. Generic Object Detection

Modern object detection methods can be broadly categorized into two-stage and one-stage paradigms. Two-stage detectors, pioneered by Faster R-CNN [4], first generate region proposals and then refine them for classification and localization. Cascade R-CNN [9] extends this pipeline with multi-stage refinement, while Sparse R-CNN [27] introduces learnable proposals to eliminate hand-crafted anchors. These methods generally achieve high accuracy but at the cost of increased computational overhead.

One-stage detectors directly predict bounding boxes and class labels from feature maps. SSD [28] employs multi-scale feature maps for detection at different resolutions. RetinaNet [5] addresses the class imbalance problem through focal loss, and FCOS [10] proposes a fully convolutional anchor-free framework. The YOLO series [11,12,13,29,30,31,32] has continuously pushed the boundary of real-time detection, with recent versions incorporating advanced training strategies, architectural innovations, and efficient feature aggregation mechanisms. Detection frameworks based on YOLO have also been successfully adapted for domain-specific applications such as agricultural pest detection [33] and building supervision [34].

### 2.2. Transformer-Based Detection

The introduction of DETR [6] marked a paradigm shift by formulating object detection as a set prediction problem using Transformers [35]. Deformable DETR [14] addresses the slow convergence of DETR through deformable attention mechanisms. DINO [36] further improves performance with contrastive denoising training and mixed query selection. RT-DETR [15] achieves real-time performance by designing an efficient hybrid encoder and uncertainty-minimal query selection. Recent works have also explored cross-attention transformers for multi-modal detection tasks [17,37], demonstrating the versatility of attention mechanisms across different sensing modalities.

Vision Transformers [38] and their hierarchical variants such as Swin Transformer [39,40] have also been widely adopted as backbone networks, providing strong feature representations through self-attention mechanisms. However, most transformer-based detectors apply uniform attention across all object categories, missing the opportunity to leverage category-specific semantic structures that are particularly important in fine-grained detection scenarios.

### 2.3. Vision-Language Priors for Visual Recognition

Vision-Language Models (VLMs) have emerged as powerful tools for encoding semantic knowledge applicable to visual recognition tasks. CLIP [21] pioneered the alignment of visual and textual representations through contrastive learning on large-scale image-text pairs, enabling zero-shot and few-shot recognition capabilities. Subsequent works have explored leveraging text embeddings as semantic priors for various vision tasks, including open-vocabulary detection and language-driven semantic segmentation [22]. Grounding DINO [41] further demonstrated the effectiveness of grounding language representations in visual detection, achieving open-set object detection by marrying transformer-based detectors with grounded pre-training.

In the remote sensing domain, recent studies have highlighted the importance of advanced recognition paradigms and robust feature learning for accurate target identification. Hou et al. [24] proposed EfficientSARNet, a lightweight yet high-performance network for SAR image target recognition, demonstrating that efficient architectural design can achieve strong recognition accuracy. Privacy-preserving federated learning has also been explored for remote sensing recognition with adaptive resource management [23], while federated clustering approaches [25] address domain shift challenges in distributed SAR recognition scenarios. Despite these advances, the potential of CLIP text priors for guiding fine-grained ship detection in optical remote sensing images remains largely unexplored. Our work bridges this gap by employing a frozen CLIP text encoder to generate category-specific semantic embeddings that provide domain-aware guidance for the detection pipeline.

### 2.4. Ship Detection in Remote Sensing

Ship detection in remote sensing images has received significant attention due to its practical importance. Early deep learning approaches adapted generic detectors to aerial images with rotated bounding box predictions. R3Det [42] proposes a refined rotation detector with feature-level alignment. Oriented R-CNN [43] generates oriented proposals efficiently, while RoI Transformer [44] learns spatial transformations for oriented objects. ReDet [45] introduces rotation-equivariant networks to handle arbitrary orientations. GDet [46] models objects as Gaussian distributions for more accurate rotation representation.

Recent ship detection methods have explored frequency-aware features [20], multi-scale feature fusion [7,47], and semantic guidance [48] to improve detection accuracy. Multi-scale detection strategies have also been explored in UAV-based remote sensing scenarios [16], and slicing-aided inference has shown clear benefits for small object detection in large images [49]. In addition, complex-environment detection and attention-enhanced remote-sensing detectors have been studied in camouflage and aerial-target scenarios [50,51]. Despite these advances, existing methods rarely integrate categorical CLIP text priors with structure-aware channel enhancement in a unified framework. SPFDet addresses this gap by combining semantic prior guidance, channel-selective transformer enhancement, and category-dominated feature fusion.

## 3. Proposed Method

### 3.1. Overall Architecture

**Figure 1:**
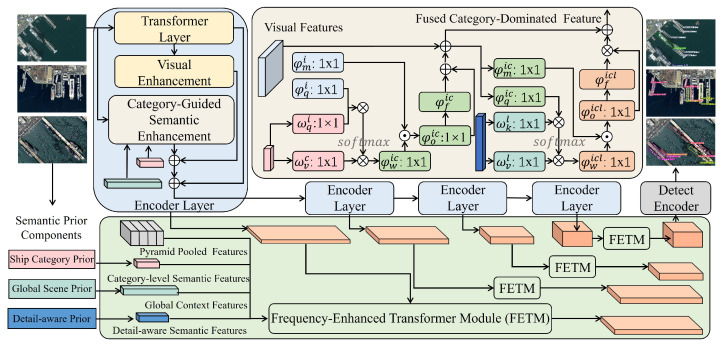
Overall architecture of SPFDet. The framework consists of three main components: Semantic Prior Component (SPC) that extracts ship category prior, global scene prior, and detail-aware prior; Structure-Enhanced Transformer Module (SETM) that enhances multi-scale encoder features through cross-level channel selection and cross-attention; and Fused Category-Dominated Feature (FCDF) generation that integrates semantic priors with visual features for category-aware detection.

The overall architecture of SPFDet is illustrated in Figure 1. Given an input remote sensing image $I\in R^{H\times W\times 3}$, SPFDet processes it through four main stages: (1) a backbone network extracts multi-scale visual features; (2) the Semantic Prior Component (SPC) generates three types of priors, where the category prior is derived from a frozen CLIP text encoder and the scene and detail priors are extracted from backbone features; (3) the Structure-Enhanced Transformer Module (SETM) enhances encoder features by selectively amplifying discriminative channel responses guided by semantic priors; and (4) the Fused Category-Dominated Feature (FCDF) generation mechanism integrates CLIP-based semantic priors with visual features to produce category-aware representations for the detection head.

Formally, the backbone network (e.g., ResNet-50 [52]) extracts a feature pyramid $\left\{C_{2},C_{3},C_{4},C_{5}\right\}$ at strides {4,8,16,32}. A Feature Pyramid Network [53] further refines these into multi-scale features $\left\{P_{2},P_{3},P_{4},P_{5}\right\}$. The encoder consists of L=4 stacked layers with feature dimension d=256. At layer *l*, the visual feature $F_{v}^{l}\in R^{H_{l}W_{l}\times d}$ is enhanced by SETM using projected semantic priors. The category prior is broadcast over spatial tokens after projection, the scene prior is repeated over all locations as global context, and the detail prior is resized to $\left(H_{l},W_{l}\right)$ by bilinear interpolation before 1×1 projection. The enhanced features are then fused by FCDF and passed to the detection head for final predictions.

### 3.2. Semantic Prior Components

The core insight behind SPC is that ship detection benefits from domain-specific semantic knowledge at multiple granularities. Rather than relying solely on data-driven feature extraction, we leverage CLIP-encoded textual knowledge to provide category-level guidance, complemented by visual priors for scene context and spatial details. We design three complementary prior extraction branches:

#### 3.2.1. Ship Category Prior

Unlike conventional approaches that derive category representations from visual features alone, we leverage a frozen CLIP text encoder [21] to generate category-level semantic embeddings from textual descriptions of ship types. For each ship category $c_{i}$ ($i=1,\ldots ,N_{c}$), we construct a descriptive text prompt $t_{i}$ that encodes the structural and functional characteristics of the category, e.g., “a remote sensing image of a destroyer, characterized by an elongated hull with visible weapon systems and radar equipment.” The frozen CLIP text encoder $\Phi_{\mathrm{text}}$ maps these prompts into a high-dimensional semantic space, and a learnable projection layer aligns the text embeddings with the visual feature space:

(1) $$F_{\mathrm{cat}}=W_{p}\cdot \Phi_{\mathrm{text}}\left(T\right)+b_{p}\in R^{N_{c}\times d_{s}},$$

where $T=\{t_{1},\ldots ,t_{N_{c}}\}$ denotes the set of category-specific text prompts, $\Phi_{\mathrm{text}}$ is the frozen CLIP text encoder that outputs embeddings in $R^{d_{t}}$, and $W_{p}\in R^{d_{s}\times d_{t}}$, $b_{p}\in R^{d_{s}}$ are learnable projection parameters. The CLIP text encoder is kept frozen during training to preserve its pre-trained vision-language semantic knowledge, while only the projection layer is optimized. The resulting $F_{\mathrm{cat}}$ encodes category-level semantic features that represent the prototypical structural patterns of each ship type, providing richer and more generalizable category representations than purely data-driven embeddings.

#### 3.2.2. Global Scene Prior

The global scene prior encodes the overall contextual information of the scene (e.g., harbor vs. open sea), which provides important cues for detection. We compute this by applying global average pooling followed by a two-layer MLP:

(2) $$F_{\mathrm{scene}}=\mathrm{MLP}\left(\mathrm{GAP}(C_{5})\right)\in R^{1\times d_{s}},$$

where GAP denotes global average pooling. The scene prior captures holistic contextual features that help distinguish challenging scenarios such as densely packed harbor scenes from open-water detection.

#### 3.2.3. Detail-Aware Prior

The detail-aware prior preserves fine-grained spatial details necessary for distinguishing visually similar ship categories. We extract it from the higher-resolution feature $C_{3}$ through a lightweight detail extraction network:

(3) $$F_{\mathrm{detail}}=\sigma \left({\mathrm{Conv}}_{3\times 3}\left({\mathrm{Conv}}_{1\times 1}\left(C_{3}\right)\right)\right)\in R^{\frac{H}{8}\times \frac{W}{8}\times d_{s}},$$

where σ denotes the GELU activation function. The detail-aware prior retains high-resolution spatial information that captures subtle structural differences (e.g., antenna configurations, deck layouts) among fine-grained ship categories.

The three priors $\left\{F_{\mathrm{cat}},F_{\mathrm{scene}},F_{\mathrm{detail}}\right\}$ are then fed into SETM at different encoder layers, providing hierarchical guidance throughout the feature enhancement process. We use “semantic prior” primarily for the CLIP category prior; the scene and detail branches are visual contextual priors that complement the text prior.

### 3.3. Structure-Enhanced Transformer Module

**Figure 2:**
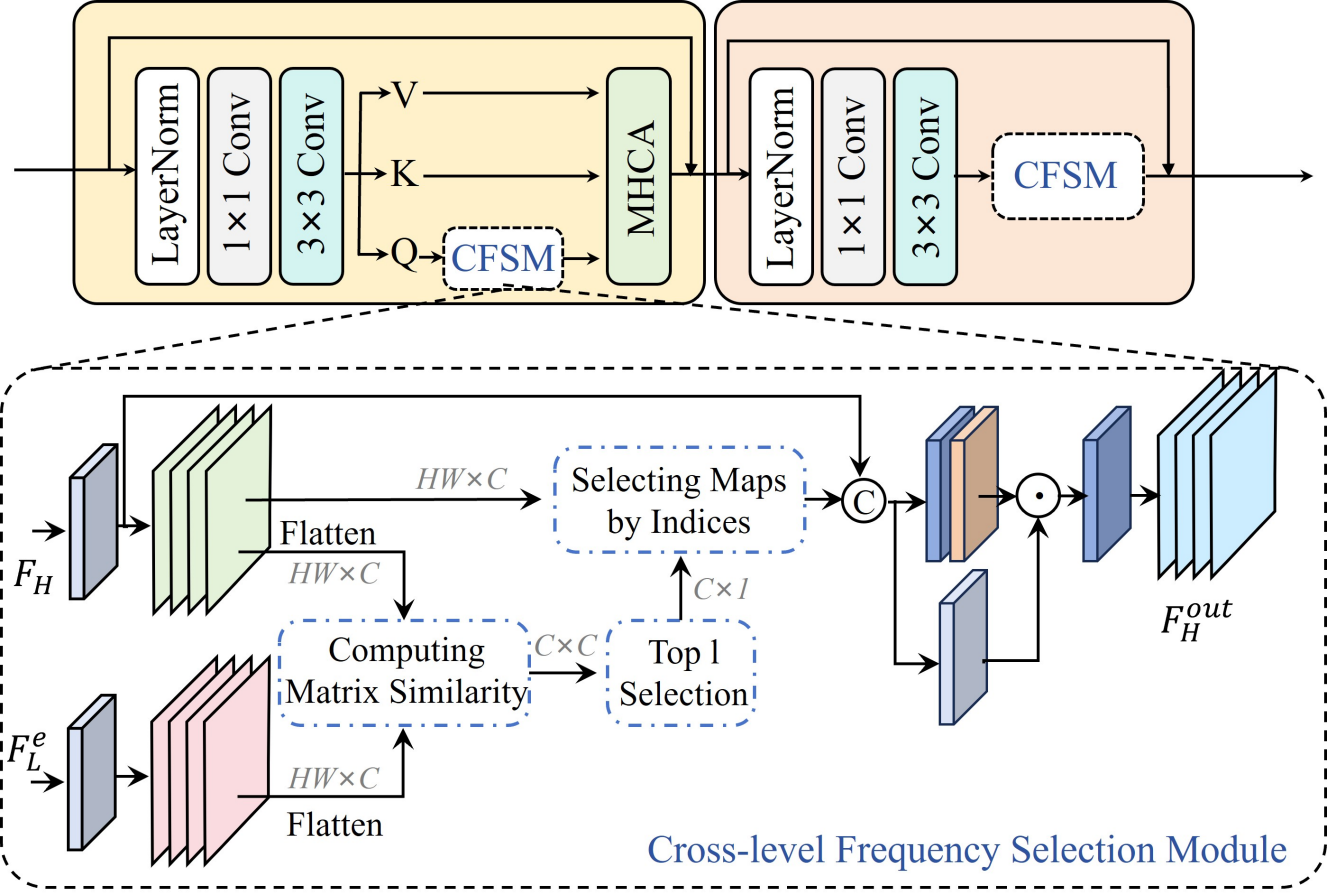
Architecture of the Structure-Enhanced Transformer Module (SETM). The upper portion shows the overall SETM block with LayerNorm, convolutions, MHCA, and CCSM. The lower portion details the Cross-level Channel Selection Module (CCSM), which selects the most relevant structural channel responses from a low-level enhanced feature $F_{L}^{e}$ to enhance the high-level feature $F_{H}$.

SETM is designed to enhance encoder features by selectively amplifying discriminative channel responses associated with ship structures. As shown in Figure 2, each SETM block consists of two sub-modules: a Cross-level Channel Selection Module (CCSM) and a Multi-Head Cross-Attention (MHCA) mechanism, connected through layer normalization and residual connections. Unlike methods that explicitly transform features into the frequency domain using FFT, DCT, or wavelets, SETM operates in the learned feature space. We therefore use the term structure-enhanced to emphasize that CCSM selects and modulates channels whose responses are correlated with hull contours, deck textures, edges, and other structural patterns.

#### 3.3.1. Cross-Level Channel Selection Module

The key motivation behind CCSM is that different learned channels respond to different structural cues: some channels emphasize the overall hull shape, while others respond to edges, decks, wakes, and fine details. Rather than using all channels equally, CCSM learns to select the most relevant correlated channels guided by cross-level feature similarity.

Given a high-level feature $F_{H}\in R^{H^{\prime }\times W^{\prime }\times C}$ from the current encoder layer and an enhanced low-level feature $F_{L}^{e}\in R^{H^{\prime }\times W^{\prime }\times C}$ from semantic priors, CCSM operates as follows. Both features are first flattened along the spatial dimension:

(4) $${\hat{F}}_{H}=\mathrm{Flatten}\left(F_{H}\right)\in R^{HW\times C},\;{\hat{F}}_{L}=\mathrm{Flatten}\left(F_{L}^{e}\right)\in R^{HW\times C}.$$

A channel-wise similarity matrix is then computed to measure the correlation between structural channel responses:

(5) $$S={\hat{F}}_{L}^{⊤}{\hat{F}}_{L}\in R^{C\times C},$$

The top-1 most similar channel index for each channel is selected:

(6) $$\mathrm{idx}=\arg\max_{j}S_{i,j}\in R^{C\times 1},\;j\neq i.$$

The selected channel maps from ${\hat{F}}_{H}$ are concatenated with the original maps and modulated through element-wise multiplication:

(7) $$F_{H}^{\mathrm{out}}={\mathrm{Conv}}_{1\times 1}\left(\mathrm{Concat}\left[{\hat{F}}_{H},{\hat{F}}_{H}\left[\mathrm{idx}\right]\right]\right)⊙{\mathrm{Conv}}_{1\times 1}\left({\hat{F}}_{H}\right)\in R^{H^{\prime }\times W^{\prime }\times C},$$

where ⊙ denotes element-wise multiplication. This mechanism amplifies the most semantically relevant structural responses while suppressing less informative or noisy channels. We use top-1 selection because it provides a deterministic one-to-one correspondence between source and target channels, avoids introducing additional Top-*K* hyperparameters, and keeps the module lightweight. In preliminary design, weighted multi-channel aggregation increased computation and sometimes smoothed discriminative fine details; therefore, the compact top-1 variant was adopted for the final model.

#### 3.3.2. Multi-Head Cross-Attention

**Figure 3:**
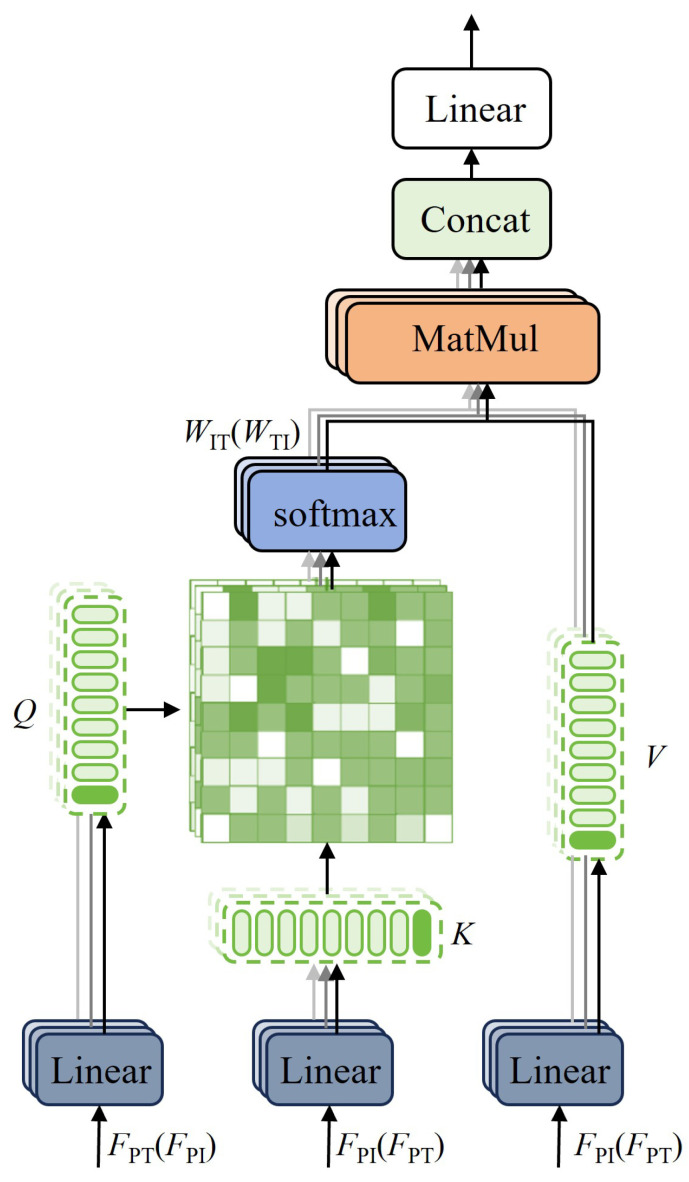
Structure of Multi-Head Cross-Attention (MHCA). The module computes bidirectional cross-attention between prior features $F_{\mathrm{PT}}$/$F_{\mathrm{PI}}$, generating attention weight matrices $W_{\mathrm{IT}}$ and $W_{\mathrm{TI}}$ for comprehensive feature interaction. The colored arrows indicate the two complementary information-flow directions and the corresponding feature-projection paths used in bidirectional cross-attention.

The MHCA module enables bidirectional information exchange between structure-enhanced visual features and semantic prior features. As illustrated in Figure 3, MHCA takes two feature inputs $F_{\mathrm{PT}}$ (prior-to-transformer) and $F_{\mathrm{PI}}$ (prior-input) and computes cross-attention in both directions. At encoder layer *l*, $F_{\mathrm{PT}}\in R^{N_{l}\times d_{s}}$ is obtained by projecting and flattening the SETM-enhanced visual feature, where $N_{l}=H_{l}W_{l}$. $F_{\mathrm{PI}}\in R^{M_{l}\times d_{s}}$ is the concatenation of projected category, scene, and detail priors, where the category and scene tokens are repeated or pooled to match the attention interface and the detail prior is resized to the current scale. If the feature dimensions differ, a 1×1 convolution or linear projection maps them to the shared dimension $d_{s}=256$.

For the forward direction (prior-to-transformer), the query, key, and value projections are:

(8) $$Q=F_{\mathrm{PT}}W_{Q},\;K=F_{\mathrm{PI}}W_{K},\;V=F_{\mathrm{PI}}W_{V},$$

where $W_{Q},W_{K},W_{V}\in R^{d_{s}\times d_{h}}$ are learnable projection matrices and $d_{h}=d_{s}/n_{h}$ is the per-head dimension with $n_{h}$ attention heads. The attention weight matrix and output are computed as:

(9) $$W_{\mathrm{IT}}={\mathrm{softmax}}_{M_{l}}\left(\frac{QK^{⊤}}{\sqrt{d_{h}}}\right),\;O_{\mathrm{IT}}=W_{\mathrm{IT}}V.$$

where ${\mathrm{softmax}}_{M_{l}}$ denotes normalization along the key/prior-token dimension. Similarly, the reverse direction (transformer-to-prior) computes $W_{\mathrm{TI}}$ and $O_{\mathrm{TI}}$ by swapping the roles of $F_{\mathrm{PT}}$ and $F_{\mathrm{PI}}$, with softmax normalization along the visual-token dimension $N_{l}$. The bidirectional outputs are concatenated and projected:

(10) $$F_{\mathrm{MHCA}}=\mathrm{Linear}\left(\mathrm{Concat}\left[O_{\mathrm{IT}},O_{\mathrm{TI}}\right]\right).$$

This bidirectional design ensures that semantic priors are not only used to modulate visual features but also enriched by visual context, creating a synergistic enhancement loop.

### 3.4. Fused Category-Dominated Feature Generation

The FCDF mechanism integrates the outputs of SETM with the original encoder features through a category-guided attention scheme. As shown in the upper-right portion of Figure 1, given the visual features from the encoder layer and the SETM-enhanced semantic features, FCDF performs the following operations.

For each encoder layer *l*, the visual feature $F_{v}^{l}\in R^{H_{l}\times W_{l}\times d}$ and the fused semantic feature $F_{s}^{l}\in R^{H_{l}\times W_{l}\times d_{s}}$ from SETM are first projected through 1×1 convolutions:

(11) $$\varphi_{m}^{i}={\mathrm{Conv}}_{1\times 1}^{m}\left(F_{v}^{l}\right),\;\varphi_{q}^{i}={\mathrm{Conv}}_{1\times 1}^{q}\left(F_{v}^{l}\right),\;\omega_{q}^{i}={\mathrm{Conv}}_{1\times 1}^{wq}\left(F_{s}^{l}\right).$$

A category-guided attention map is computed through softmax normalization:

(12) $$\varphi_{f}^{ic}=\mathrm{softmax}\left(\varphi_{q}^{i}⊗\omega_{q}^{i}\right),$$

where ⊗ denotes the outer product operation. The intermediate fused feature is obtained by: Here, the visual tensor is flattened to $N_{l}=H_{l}W_{l}$ spatial tokens before attention. The softmax in Equation (12) is applied along the semantic/category-token dimension so that each spatial token receives normalized category-prior weights.

(13) $$\varphi_{o}^{ic}=\omega_{v}^{c}⊗\varphi_{f}^{ic}⊕\varphi_{m}^{i},$$

where $\omega_{v}^{c}={\mathrm{Conv}}_{1\times 1}^{wv}\left(F_{s}^{l}\right)$ and ⊕ denotes element-wise addition. This process is repeated at the second stage to produce the final Fused Category-Dominated Feature:

(14) $$F_{\mathrm{FCDF}}^{l}=\varphi_{w}^{icl}⊗\mathrm{softmax}\left(\varphi_{q}^{ic}⊗\omega_{k}^{l}\right)⊕\varphi_{m}^{ic},$$

where $\varphi_{m}^{ic},\varphi_{q}^{ic},\omega_{k}^{l}$ are projections of the intermediate features and semantic features at subsequent levels. The second-stage softmax is also normalized along the key-token dimension. After attention, the output is reshaped back to $R^{H_{l}\times W_{l}\times d}$ and aligned with the original visual feature by residual addition. The multi-scale FCDF features ${\left\{F_{\mathrm{FCDF}}^{l}\right\}}_{l=1}^{L}$ are then passed to the detection encoder for final bounding box regression and classification.

### 3.5. Loss Function

SPFDet employs a multi-task loss consisting of three components: classification loss, regression loss, and a novel Category-Semantic Consistency (CSC) loss. The overall training procedure is summarized in Algorithm 1.

Classification Loss. We adopt the focal loss [5] to handle the class imbalance between foreground ship instances and background regions:

(15) $$L_{\mathrm{cls}}=-\frac{1}{N_{\mathrm{pos}}}\sum_{i=1}^{N}\alpha_{t}{\left(1-p_{t}\right)}^{\gamma }\log\left(p_{t}\right),$$

where $p_{t}$ is the predicted probability for the ground-truth class, $\alpha_{t}$ is the balancing factor, and γ is the focusing parameter.
**Algorithm 1** Training Procedure of SPFDet**Require:** 
Training set $D={\left\{\left(I_{k},G_{k}\right)\right\}}_{k=1}^{K}$, backbone $\Phi_{b}$, frozen CLIP text encoder $\Phi_{\mathrm{text}}$, SPC module $\Phi_{s}$, SETM module $\Phi_{f}$, FCDF module $\Phi_{c}$, detection head $\Phi_{d}$, text prompts T, learning rate η, total epochs *T***Ensure:** 
Trained model parameters $\Theta^{*}$  1:Initialize model parameters Θ with pretrained backbone weights  2:$F_{\mathrm{cat}}\leftarrow W_{p}\cdot \Phi_{\mathrm{text}}\left(T\right)+b_{p}$                                                ▹ Pre-compute CLIP text-prior embeddings  3:**for** epoch =1 to *T* **do**  4:    **for** each mini-batch (I,G)∈D **do**  5:        ${\left\{C_{l}\right\}}_{l=2}^{5}\leftarrow \Phi_{b}\left(I\right)$                                                                                   ▹ Extract backbone features  6:        ${\left\{P_{l}\right\}}_{l=2}^{5}\leftarrow \mathrm{FPN}\left(\left\{C_{l}\right\}\right)$                                                                     ▹ Feature pyramid construction  7:        $F_{\mathrm{scene}},F_{\mathrm{detail}}\leftarrow \Phi_{s}\left(\left\{C_{l}\right\}\right)$                                                                            ▹ Visual prior extraction  8:        **for** each encoder layer l=1 to *L* **do**  9:           $F_{v}^{l}\leftarrow {\mathrm{EncoderLayer}}_{l}\left(P_{l}\right)$                                                                      ▹ Visual feature encoding10:           $F_{\mathrm{enh}}^{l}\leftarrow \Phi_{f}\left(F_{v}^{l},F_{\mathrm{cat}},F_{\mathrm{scene}},F_{\mathrm{detail}}\right)$                                                                 ▹ SETM enhancement11:           $F_{\mathrm{FCDF}}^{l}\leftarrow \Phi_{c}\left(F_{v}^{l},F_{\mathrm{enh}}^{l}\right)$                                                                                       ▹ FCDF generation12:        **end for**13:        $\hat{P}\leftarrow \Phi_{d}\left(\left\{F_{\mathrm{FCDF}}^{l}\right\}\right)$                                                                                        ▹ Detection predictions14:        Compute $L_{\mathrm{cls}}$, $L_{\mathrm{reg}}$, $L_{\mathrm{CSC}}$ via Equations (15)–(17)15:        $L\leftarrow \lambda_{\mathrm{cls}}L_{\mathrm{cls}}+\lambda_{\mathrm{reg}}L_{\mathrm{reg}}+\lambda_{\mathrm{CSC}}L_{\mathrm{CSC}}$16:        $\Theta \leftarrow \Theta -\eta \nabla_{\Theta }L$                                                                                               ▹ Update parameters17:    **end for**18:    Adjust learning rate with cosine annealing schedule19:**end for**20:**return**$\Theta^{*}\leftarrow \Theta$

Regression Loss. The bounding box regression loss combines the $\ell_{1}$ loss and Generalized IoU loss [54] for robust localization. For HRSC2016, SPFDet predicts oriented bounding boxes represented as $b=(x_{c},y_{c},w,h,\theta )$, where $\left(x_{c},y_{c}\right)$ is the box center, *w* and *h* are width and height, and θ is the rotation angle. The IoU term is computed using rotated box overlap, and the GIoU formulation follows the minimum enclosing rotated rectangle used in MMRotate. For ShipRSImageNet, the same detection head is used with the dataset’s bounding-box annotation format.

(16) $$L_{\mathrm{reg}}=\frac{1}{N_{\mathrm{pos}}}\sum_{i=1}^{N_{\mathrm{pos}}}\left[\lambda_{L1}{∥{\hat{b}}_{i}-b_{i}∥}_{1}+\lambda_{\mathrm{GIoU}}L_{\mathrm{GIoU}}\left({\hat{b}}_{i},b_{i}\right)\right],$$

where ${\hat{b}}_{i}$ and $b_{i}$ denote the predicted and ground-truth bounding boxes, respectively. We set $\lambda_{L1}=5.0$ and $\lambda_{\mathrm{GIoU}}=2.0$ in all experiments.

Category-Semantic Consistency Loss. To enforce alignment between the predicted detection features and the semantic priors from SPC, we introduce a CSC loss based on cosine similarity:

(17) $$L_{\mathrm{CSC}}=1-\frac{1}{N_{\mathrm{pos}}}\sum_{i=1}^{N_{\mathrm{pos}}}\frac{e_{i}^{\mathrm{pred}}\cdot e_{c_{i}}^{\mathrm{prior}}}{∥e_{i}^{\mathrm{pred}}∥\cdot ∥e_{c_{i}}^{\mathrm{prior}}∥},$$

where $e_{i}^{\mathrm{pred}}\in R^{d_{s}}$ is the category embedding extracted from the FCDF feature at the query assigned to the *i*-th positive prediction, $e_{c_{i}}^{\mathrm{prior}}\in R^{d_{s}}$ is the corresponding semantic prior embedding for ground-truth class $c_{i}$, and the summation runs over all $N_{\mathrm{pos}}$ positive matches. Positive matches are determined by the same Hungarian assignment used by the DETR-style detection head, with classification, box regression, and IoU costs. This loss encourages the model to learn detection features that are semantically consistent with the category priors, improving fine-grained classification accuracy.

Total Loss. The overall training objective is:

(18) $$L=\lambda_{\mathrm{cls}}L_{\mathrm{cls}}+\lambda_{\mathrm{reg}}L_{\mathrm{reg}}+\lambda_{\mathrm{CSC}}L_{\mathrm{CSC}},$$

where $\lambda_{\mathrm{cls}}$, $\lambda_{\mathrm{reg}}$, and $\lambda_{\mathrm{CSC}}$ are balancing coefficients set to 2.0, 5.0, and 1.0, respectively.

## 4. Experiments

### 4.1. Datasets

We evaluate SPFDet on two widely-used ship detection benchmarks:

HRSC2016 [26] is a high-resolution ship collection dataset containing 1061 images collected from Google Earth, with resolutions ranging from 300×300 to 1500×900 pixels. The dataset focuses on single-class ship detection with oriented bounding box annotations. Following standard practice, we use 436 images for training and 444 images for testing, and report mean Average Precision (mAP) under both VOC2007 and VOC2012 evaluation protocols.

ShipRSImageNet [8] is a large-scale fine-grained ship detection dataset comprising 3435 images with 17,573 ship instances across 50 categories organized in a four-level hierarchy. We evaluate on the Level-3 categorization with 20 classes including Warship, Cargo, Cruiser, Submarine, Destroyer, and others. The official split provides 2507 training images and 928 testing images. We report mAP at IoU thresholds of 0.5 (mAP_50_), 0.75 (mAP_75_), and 0.5:0.95 (mAP).

### 4.2. Implementation Details

SPFDet is implemented based on PyTorch (version 2.1.0), MMDetection (version 3.2.0), and MMRotate (version 1.0.0) [55]. We adopt ResNet-50 [52] pretrained on ImageNet as the backbone. The encoder consists of L=4 layers with d=256 feature channels. The semantic embedding dimension $d_{s}$ is set to 256, and MHCA uses $n_{h}=8$ attention heads. For the CLIP-based category prior, we employ the CLIP ViT-B/32 text encoder [21] (output dimension $d_{t}=512$), which is kept frozen throughout training. Category-specific text prompts are manually designed with structural descriptions for each ship type (e.g., “a remote sensing image of a submarine, characterized by a streamlined cylindrical hull with a conning tower”). Only the projection layer ($W_{p}$, $b_{p}$) is trainable, adding negligible parameters (∼0.13 M). Input images are resized to 800×800 for HRSC2016 and 1024×1024 for ShipRSImageNet.

We train the model using the AdamW optimizer [56] with an initial learning rate of $1\times 10^{-4}$, weight decay of $1\times 10^{-4}$, and batch size of 8. A cosine annealing schedule is employed over 120 epochs for HRSC2016 and 72 epochs for ShipRSImageNet. The focal loss parameters are set to α=0.25 and γ=2.0. Data augmentation includes random horizontal flipping, random rotation (±90°), and multi-scale training. Unless otherwise stated, $\lambda_{\mathrm{cls}}=2.0$, $\lambda_{\mathrm{reg}}=5.0$, $\lambda_{\mathrm{CSC}}=1.0$, $\lambda_{L1}=5.0$, and $\lambda_{\mathrm{GIoU}}=2.0$. All experiments are conducted on 4 NVIDIA A100 GPUs with 80 GB memory, and random seeds are fixed for dataset splitting and model initialization. FPS is measured on a single A100 GPU with batch size 1 after 50 warm-up iterations and 500 timed iterations, using FP32 inference and including model forward propagation and post-processing but excluding image loading from disk.

Prompt design. To reduce prompt-dependent variation, all category prompts follow the same template: “a remote sensing image of a [ship category], characterized by [category-specific structural attributes].” The category name anchors the semantic concept, while the attribute phrase describes observable geometry, deck layout, and functional components that are visible from overhead imagery. Table 1 lists the prompts used for the 20-class ShipRSImageNet Level-3 setting. For HRSC2016, which is a single-class ship detection dataset, the category text prior mainly provides generic ship-shape guidance for background suppression and oriented localization rather than fine-grained classification.

Baseline reporting. For reproduced methods, we use the same dataset split, input resolution, training schedule, augmentation strategy, and evaluation protocol as SPFDet whenever the original implementation permits. Results not marked with † are taken from the corresponding papers or official reports under the closest available setting, and the backbone reported in the table is kept consistent with the cited source. All FPS values in our tables are measured under the protocol above when reproduced; literature FPS values are retained only when reproduction code or checkpoints are unavailable.

### 4.3. Comparison with State-of-the-Art

#### 4.3.1. Results on HRSC2016

Table 2 presents comparisons with 17 state-of-the-art methods on HRSC2016, organized by detector category. SPFDet achieves the highest mAP under both VOC2007 (93.8%) and VOC2012 (97.2%) protocols among the compared methods.

Among two-stage detectors, Oriented R-CNN achieves strong performance (90.5%/96.5%) due to its oriented proposal generation, and SPFDet further improves the VOC2007/VOC2012 results by 3.3%/0.7%. Among one-stage methods, R3Det attains competitive 89.3%/96.1% through its rotation refinement mechanism. YOLO-based detectors show progressively improving performance with YOLOv9-C reaching 90.2%/90.7%. Among transformer-based approaches, RT-DETR-L achieves 91.2%/96.8% with its hybrid encoder design. These results indicate that semantic prior guidance and structure-aware channel enhancement are beneficial for ship detection, while the improvement on HRSC2016 is relatively moderate under the VOC2012 protocol.

**Figure 4:**
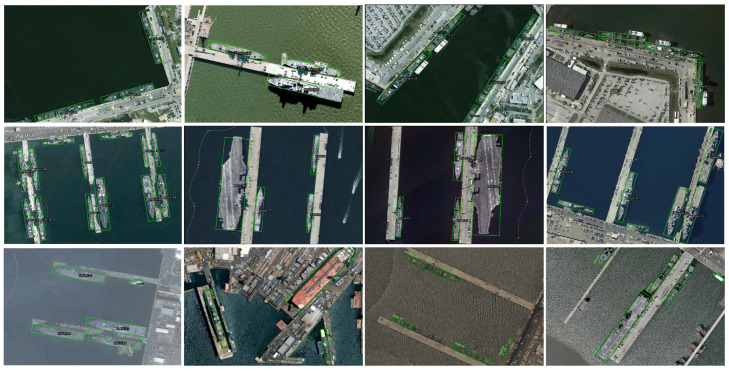
Qualitative detection results of SPFDet on the HRSC2016 dataset. The green bounding boxes indicate detected ships. Our method successfully handles challenging scenarios including dense ship clusters, varying scales, and complex harbor backgrounds.

Figure 4 presents qualitative detection results on HRSC2016, showing that SPFDet accurately localizes ships across diverse scenarios including densely packed harbors, isolated vessels in open water, and ships with varying orientations and scales.

#### 4.3.2. Results on ShipRSImageNet

Table 3 reports results on the more challenging ShipRSImageNet dataset with 20 fine-grained ship categories. SPFDet achieves the best performance across all metrics with 72.8% mAP_50_, 48.5% mAP_75_, and 43.2% mAP, demonstrating strong fine-grained classification capability.

The performance gains on ShipRSImageNet are more pronounced than on HRSC2016, which is expected since the fine-grained multi-class nature of ShipRSImageNet better demonstrates the advantages of our semantic prior guidance and category-dominated feature fusion. Compared to the second-best RT-DETR-L, SPFDet improves mAP_50_ by 3.5%, mAP_75_ by 2.7%, and mAP by 1.7%, highlighting the effectiveness of category-aware detection.

**Figure 5:**
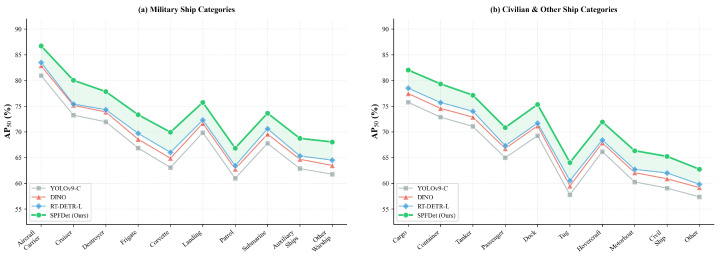
Per-category AP_50_ (%) comparison on ShipRSImageNet. (**a**) Military ship categories. (**b**) Civilian and other ship categories. The green shaded area highlights the performance margin of SPFDet over the second-best method. Our method achieves consistent improvements across all 20 categories, with pronounced gains on fine-grained military types and data-scarce civilian categories.

To further analyze the per-category performance, Figure 5 presents the AP_50_ of each ship category for four representative methods. SPFDet consistently outperforms all competitors across every category. The improvements are particularly pronounced for challenging categories with fewer training samples (e.g., Tug, Other, Civil Ship) and categories with high inter-class similarity (e.g., Cruiser vs. Destroyer, Frigate vs. Corvette), confirming that our semantic prior guidance effectively enhances fine-grained discrimination.

**Figure 6:**
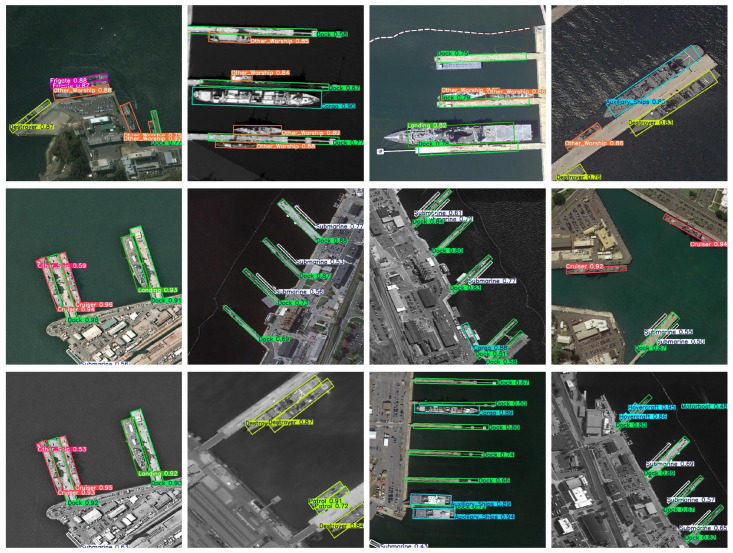
Qualitative detection results of SPFDet on the ShipRSImageNet dataset. Different colors represent different ship categories. Our method demonstrates strong fine-grained classification capability across diverse ship types including Cruiser, Destroyer, Submarine, Landing, and Cargo ships.

Figure 6 illustrates detection results on ShipRSImageNet, where SPFDet correctly classifies diverse ship types including Cruiser, Destroyer, Submarine, Cargo, and Dock ships with high confidence scores, even in cluttered harbor environments with multiple ship types.

**Figure 7:**
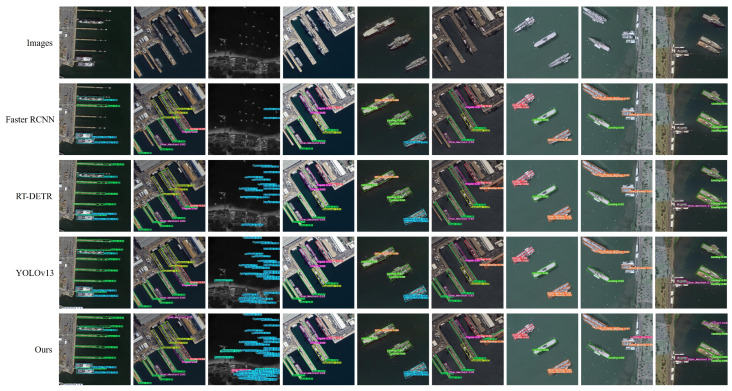
Visual comparison of detection results on ShipRSImageNet between different methods. From top to bottom: original images, Faster R-CNN [4], RT-DETR [15], YOLOv9-C [13], and SPFDet (Ours). Our method achieves accurate classification and localization with fewer false alarms in these examples.

Figure 7 provides a visual comparison between SPFDet and representative methods from different categories (Faster R-CNN, RT-DETR, and YOLOv9-C). Our method produces accurate bounding boxes with fewer missed detections and false positives in many small and densely arranged ship scenes.

### 4.4. Ablation Studies

#### 4.4.1. Effectiveness of Each Component

To validate the contribution of each proposed component, we conduct ablation experiments on ShipRSImageNet by progressively adding components to a baseline detector (Deformable DETR with ResNet-50 backbone). The results are summarized in Table 4.

Adding SPC alone improves mAP_50_ by 2.1%, confirming that semantic priors provide valuable guidance. SETM further boosts performance by 2.5%, demonstrating the effectiveness of structure-aware channel enhancement. MHCA contributes an additional 2.2% improvement through bidirectional cross-attention. Finally, FCDF brings another 2.2% gain by generating category-dominated features. The cumulative improvement of 9.0% mAP_50_ suggests that the components contribute synergistically.

#### 4.4.2. Impact of Loss Components

Table 5 examines the contribution of each loss component. Removing the CSC loss causes a notable 1.8% drop in mAP_50_, confirming that semantic consistency supervision is essential for fine-grained classification. Using only $\ell_{1}$ or GIoU for regression yields suboptimal results, while their combination provides the best localization accuracy.

**Figure 8:**
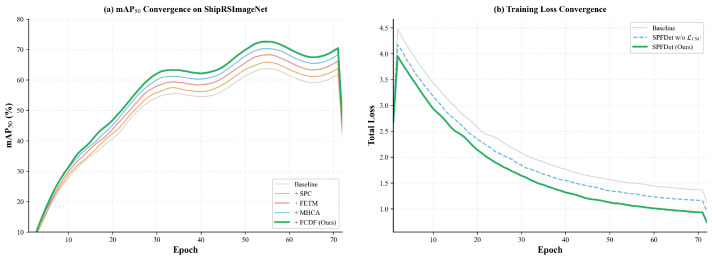
Training convergence analysis on ShipRSImageNet. (**a**) mAP_50_ convergence curves for progressive component additions, demonstrating that each component contributes to higher final performance with stable training. (**b**) Total loss convergence comparison, showing that the Category-Semantic Consistency loss ($L_{\mathrm{CSC}}$) enables faster and more stable convergence.

To further understand the training dynamics, Figure 8 visualizes the convergence behavior of different model configurations. As shown in Figure 8a, progressively adding components consistently improves the final mAP while maintaining stable convergence. The full SPFDet converges smoothly to the highest mAP_50_ of 72.8%. Figure 8b compares the total training loss, showing that the CSC loss contributes to faster and more stable convergence by providing additional semantic supervision signals.

#### 4.4.3. Impact of Semantic Prior Types

Table 6 investigates the contribution of each semantic prior type. Using only the ship category prior already provides a 1.5% improvement over the baseline without priors, validating our hypothesis that category-level patterns are important for ship detection. Adding the global scene prior further improves performance by helping the model contextualize detection scenarios. The detail-aware prior contributes the most among individual priors on the fine-grained mAP metric, as it preserves spatial details crucial for distinguishing similar ship types.

#### 4.4.4. Impact of Category Prior Generation Strategies

A key design choice in SPFDet is the use of a frozen CLIP text encoder for generating category-level semantic priors. To validate this choice, Table 7 compares different strategies for generating the ship category prior $F_{\mathrm{cat}}$ while keeping all other components identical.

Several observations can be drawn. First, random embeddings yield the lowest performance, confirming that meaningful category representations are essential. Second, learned embeddings and PPF-based visual priors achieve comparable results (71.0% vs. 71.3% mAP_50_), but both are limited by the training data distribution. Third, among language model-based approaches, Word2Vec provides marginal improvement due to its limited contextual understanding, while BERT offers stronger performance through its contextualized representations. Finally, the CLIP text encoder achieves the best results, outperforming the learned embedding baseline by 1.8% mAP_50_ and 1.2% mAP. We attribute this superiority to CLIP’s vision-language alignment pre-training, which produces text embeddings that are inherently compatible with visual feature spaces, facilitating more effective cross-modal semantic guidance.

#### 4.4.5. Visualization Analysis

**Figure 9:**
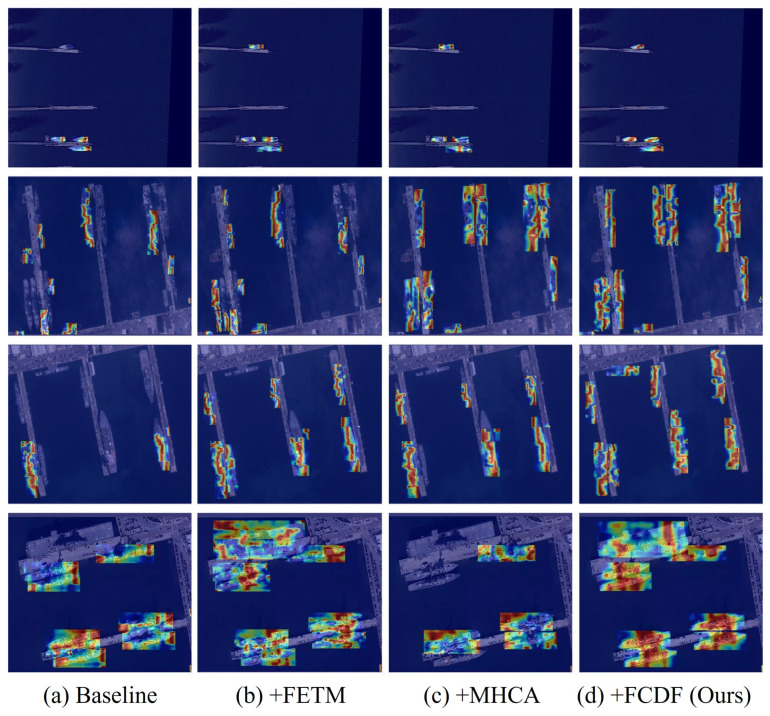
Grad -CAM visualization of ablation configurations on ShipRSImageNet. (**a**) Baseline shows diffuse activation. (**b**) +SETM produces more concentrated attention on ship structures. (**c**) +MHCA further refines discriminative regions. (**d**) +FCDF (Ours, full SPFDet) produces focused attention around ship boundaries and distinctive features.

To provide deeper insights into how each component contributes to detection, we visualize Grad-CAM [57] activation maps for the ablation configurations in Figure 9. The baseline model produces diffuse attention that often extends to background regions. Adding SETM concentrates attention more precisely on ship regions by amplifying relevant structural channel responses. MHCA further refines the attention to focus on discriminative parts of ships. The full SPFDet (with FCDF) achieves concentrated attention maps that highlight ship boundaries and internal structures while suppressing background distractions.

These visualizations indicate that: (1) SETM enhances channel responses corresponding to ship structural patterns; (2) MHCA enables the model to attend to discriminative ship regions through semantic-guided cross-attention; and (3) FCDF integrates these enhancements into category-dominated representations for ship instances.

#### 4.4.6. Computational Efficiency Analysis

Table 8 reports the computational costs of SPFDet compared with representative methods. Despite incorporating additional modules, SPFDet maintains reasonable computational overhead with 38.7M parameters and 156.3 GFLOPs, comparable to RT-DETR-L while achieving higher accuracy in our setting. The inference speed of 35.6 FPS suggests practical potential for offline or near-real-time remote sensing analysis, although deployment speed may vary across hardware platforms.

## 5. Discussion

The experimental results demonstrate that SPFDet consistently outperforms state-of-the-art methods on both HRSC2016 and ShipRSImageNet benchmarks. We attribute this success to several factors and discuss key observations:

CLIP text priors help bridge the domain gap. Generic object detectors treat all categories uniformly, missing the opportunity to exploit domain-specific knowledge. Our SPC module introduces ship-specific inductive biases through CLIP category embeddings, visual scene priors, and detail-aware priors. The ablation study on category prior generation strategies (Table 7) shows that CLIP text embeddings outperform learned embeddings and purely visual priors in our setting, suggesting that vision-language pre-training provides useful category representations for fine-grained ship detection. The ablation study on prior types (Table 6) further shows that combining all three priors yields the best performance, as they capture complementary aspects of ship semantics. This finding aligns with recent observations in multi-modal remote sensing detection [16] and intelligent recognition [18], where incorporating external knowledge and domain priors consistently improves accuracy.

Channel selection enhances structural discrimination. Ships possess distinctive structural patterns such as hull shapes, deck configurations, and superstructures. CCSM selectively amplifies correlated structural channel responses rather than processing all channels uniformly. The Grad-CAM visualizations (Figure 9) indicate that SETM focuses attention on ship structures, producing sharper feature responses compared to the baseline.

Cross-attention enables semantic-visual synergy. The bidirectional design of MHCA creates a mutual enhancement loop: semantic priors guide visual feature refinement, while visual context enriches prior representations. This is particularly effective for handling challenging cases such as partially occluded ships or ships in unusual poses, where either semantic or visual information alone may be insufficient.

Category-dominated features improve fine-grained classification. The largest performance gains are observed on ShipRSImageNet with its 20 fine-grained categories, where SPFDet improves over the second-best method by 3.5% mAP_50_. This demonstrates that the FCDF mechanism effectively generates category-discriminative features. In contrast, on the single-class HRSC2016, the gains are relatively smaller since fine-grained classification is not required; the semantic prior mainly helps background suppression and structure-aware localization.

**Figure 10:**
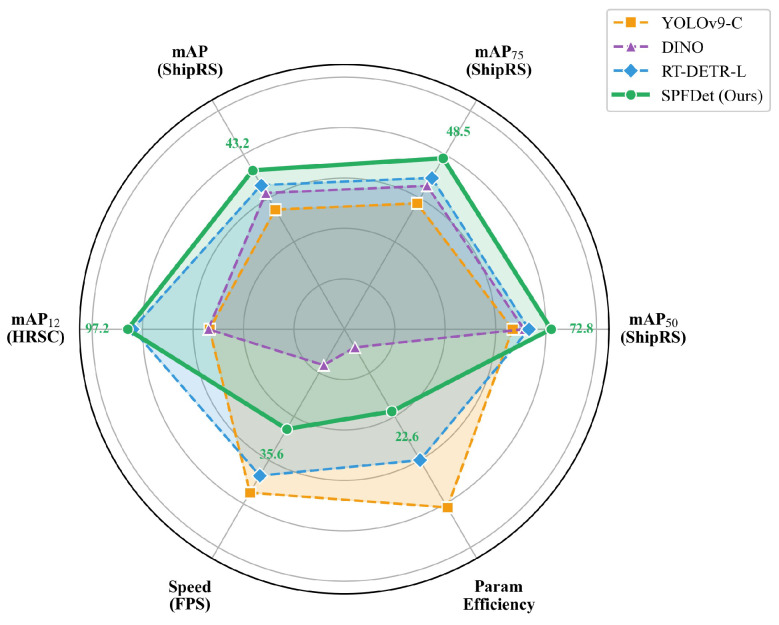
Multi-dimensional performance comparison among top-performing methods via radar chart. Each axis is min-max normalized among the compared methods, and parameter efficiency uses the inverse normalized parameter count. SPFDet (green, solid) provides a balanced accuracy-efficiency trade-off across detection accuracy, HRSC2016 mAP_12_, inference speed, and parameter efficiency.

Multi-dimensional comparison. To provide a holistic comparison, Figure 10 presents a radar chart evaluating SPFDet against three top-performing competitors across six dimensions: mAP_50_, mAP_75_, and mAP on ShipRSImageNet, mAP(VOC12) on HRSC2016, inference speed (FPS), and parameter efficiency. Each axis is min-max normalized among the compared methods, and parameter efficiency is computed as the inverse normalized parameter count so that a larger value consistently indicates a more favorable result. SPFDet obtains a large coverage area because it improves the accuracy metrics while maintaining competitive speed and model size. RT-DETR-L achieves higher FPS, YOLOv9-C has strong speed and parameter efficiency, and DINO provides competitive accuracy; SPFDet offers a balanced accuracy-efficiency trade-off in our experimental setting.

Limitations and future work. While SPFDet achieves strong performance, several directions warrant future exploration. First, the current text prompts are manually designed, and performance may be affected by prompt wording. Future work will investigate prompt ensembling, automatic prompt optimization, and stronger text encoders under controlled parameter budgets. Second, SETM does not perform an explicit FFT, DCT, or wavelet transform; it enhances learned structural channel responses. Introducing explicit spectral transforms may further improve frequency-sensitive representation. Third, the model focuses on optical remote sensing images; extending to SAR (Synthetic Aperture Radar) imagery would broaden its applicability, where recent advances in efficient SAR recognition [24] and privacy-preserving federated learning for SAR targets [23,25] provide promising complementary techniques. Finally, recent advances in lightweight model design [58] and efficient detection architectures [32] suggest opportunities for reducing computational overhead while maintaining accuracy.

## 6. Conclusions

In this paper, we proposed SPFDet, a CLIP text-prior-guided structure-enhanced detector for fine-grained ship detection in remote sensing images. By leveraging a frozen CLIP text encoder to generate category-level semantic embeddings from textual descriptions of ship types and combining them with visual scene and detail-aware priors, SPFDet addresses the challenges of complex backgrounds, scale variations, and fine-grained inter-class similarities. The Structure-Enhanced Transformer Module with cross-level channel selection and multi-head cross-attention selectively amplifies discriminative structural responses guided by semantic priors, while the Fused Category-Dominated Feature generation mechanism integrates these priors with multi-scale visual features through category-guided attention for precise detection. Extensive experiments on HRSC2016 and ShipRSImageNet benchmarks demonstrate competitive state-of-the-art performance with 97.2% and 72.8% mAP respectively among the compared methods. Ablation studies confirm the benefit of CLIP-based semantic priors over conventional learned embeddings (1.8% mAP_50_ improvement) and validate the contribution of each component through quantitative analysis and Grad-CAM visualizations.

## Figures and Tables

**Table 1:** CLIP text prompts used to generate category priors for ShipRSImageNet Level-3.

Category	Prompt
Warship	a remote sensing image of a warship, characterized by an elongated hull, deck weapons, and radar equipment
Aircraft carrier	a remote sensing image of an aircraft carrier, characterized by a flat flight deck and island superstructure
Cruiser	a remote sensing image of a cruiser, characterized by a long combat hull and multiple radar or missile systems
Destroyer	a remote sensing image of a destroyer, characterized by an elongated hull with visible weapon systems and radar equipment
Frigate	a remote sensing image of a frigate, characterized by a compact escort hull and central superstructure
Corvette	a remote sensing image of a corvette, characterized by a small patrol hull and compact deck equipment
Submarine	a remote sensing image of a submarine, characterized by a streamlined cylindrical hull with a conning tower
Landing ship	a remote sensing image of a landing ship, characterized by a broad deck and ramp-like bow structure
Patrol ship	a remote sensing image of a patrol ship, characterized by a narrow hull and lightweight deck facilities
Auxiliary ship	a remote sensing image of an auxiliary ship, characterized by support equipment and a service-oriented deck layout
Cargo ship	a remote sensing image of a cargo ship, characterized by a large rectangular cargo area and long hull
Container ship	a remote sensing image of a container ship, characterized by stacked container blocks and a regular deck grid
Tanker	a remote sensing image of a tanker, characterized by a long smooth deck and cylindrical storage layout
Bulk carrier	a remote sensing image of a bulk carrier, characterized by large cargo hatches distributed along the deck
Fishing boat	a remote sensing image of a fishing boat, characterized by a small hull and visible working deck equipment
Tug	a remote sensing image of a tug boat, characterized by a compact hull and strong towing superstructure
Engineering ship	a remote sensing image of an engineering ship, characterized by cranes or construction equipment on deck
Dock ship	a remote sensing image of a dock ship, characterized by a large open well deck or loading structure
Civil ship	a remote sensing image of a civil ship, characterized by non-military deck layout and transport-oriented structure
Other ship	a remote sensing image of another ship type, characterized by generic hull contours and visible deck structures

**Table 2:** Comparison with state-of-the-art methods on HRSC2016. The best results are in **bold** and the second best are underlined. † denotes results reproduced by us.

Category	Method	Backbone	mAP (%, 07)	mAP (%, 12)	Params (M)
Two-Stage	Faster R-CNN [4]	ResNet-50	84.6	85.3	41.5
	Cascade R-CNN [9]	ResNet-50	87.2	88.0	69.2
	Sparse R-CNN [27]	ResNet-50	86.5	87.1	106.1
	Oriented R-CNN [43]	ResNet-50	90.5	96.5	41.1
	RoI Transformer [44]	ResNet-50	86.2	87.4	55.3
One-Stage	SSD [28] ^†^	VGG-16	79.5	80.8	26.3
	RetinaNet [5]	ResNet-50	82.9	84.3	36.5
	FCOS [10]	ResNet-50	84.1	85.6	32.1
	R3Det [42]	ResNet-50	89.3	96.1	41.9
YOLO-Based	YOLOX-L [30] ^†^	CSPDarknet	87.8	88.5	54.2
	YOLOv7 [11] ^†^	E-ELAN	88.5	89.2	37.2
	YOLOv8-L [12] ^†^	CSPDarknet	89.6	90.1	43.6
	YOLOv9-C [13] ^†^	GELAN	90.2	90.7	25.5
Transformer	DETR [6]	ResNet-50	83.5	84.2	41.3
	Deformable DETR [14]	ResNet-50	87.3	88.6	40.0
	DINO [36]	ResNet-50	90.1	90.8	47.5
	RT-DETR-L [15]	HGNetv2	91.2	96.8	32.0
	**SPFDet (Ours)**	ResNet-50	**93.8**	**97.2**	38.7

**Table 3:** Comparison with state-of-the-art methods on ShipRSImageNet (Level-3, 20 classes). The best results are in **bold** and the second best are underlined.

Category	Method	mAP_50_ (%)	mAP_75_ (%)	mAP (%)	FPS
Two-Stage	Faster R-CNN [4]	58.3	34.7	32.1	18.2
	Cascade R-CNN [9]	61.5	37.8	34.6	14.5
	Sparse R-CNN [27]	60.2	36.3	33.5	22.8
	Oriented R-CNN [43]	65.8	41.2	37.9	16.7
	ReDet [45]	66.3	42.0	38.5	13.2
One-Stage	SSD [28]	48.7	26.3	24.8	62.5
	RetinaNet [5]	55.4	32.1	29.8	32.1
	FCOS [10]	57.8	33.9	31.5	35.4
	R3Det [42]	63.7	39.5	36.2	21.5
YOLO-Based	YOLOX-L [30]	61.2	37.4	34.3	52.6
	YOLOv7 [11]	63.5	39.1	35.8	55.8
	YOLOv8-L [12]	65.1	40.6	37.2	58.3
	YOLOv9-C [13]	66.7	42.3	38.7	48.7
Transformer	DETR [6]	54.6	31.2	28.7	28.3
	Deformable DETR [14]	63.8	40.1	36.8	25.6
	DINO [36]	68.5	44.7	40.6	22.4
	RT-DETR-L [15]	69.3	45.8	41.5	45.2
	**SPFDet (Ours)**	**72.8**	**48.5**	**43.2**	35.6

**Table 4:** Ablation study on the effectiveness of each component on ShipRSImageNet. Baseline: Deformable DETR with ResNet-50.

Configuration	SPC	SETM	MHCA	FCDF	mAP_50_ (%)	mAP (%)
Baseline					63.8	36.8
+ SPC	✓				65.9	38.3
+ SETM	✓	✓			68.4	40.1
+ MHCA	✓	✓	✓		70.6	41.8
+ FCDF (SPFDet)	✓	✓	✓	✓	**72.8**	**43.2**

**Table 5:** Ablation study on loss components on ShipRSImageNet.

L _cls_	$L_{L1}$	L _GIoU_	L _CSC_	mAP_50_ (%)	mAP (%)
✓	✓			69.5	40.3
✓		✓		70.1	41.0
✓	✓	✓		71.0	41.9
✓	✓	✓	✓	**72.8**	**43.2**

**Table 6:** Ablation study on semantic prior types on ShipRSImageNet.

Category Prior	Scene Prior	Detail Prior	mAP_50_ (%)	mAP (%)
			69.8	41.0
✓			71.3	42.2
	✓		71.0	42.0
		✓	71.4	42.5
✓	✓		71.8	42.6
✓		✓	72.1	42.8
✓	✓	✓	**72.8**	**43.2**

**Table 7:** Ablation study on category prior generation strategies on ShipRSImageNet. “Learned Emb.” denotes randomly initialized learnable category embeddings optimized during training. “PPF” denotes Pyramid Pooled Features extracted from backbone feature $C_{5}$.

Prior Source	Text Encoder	Frozen	mAP_50_ (%)	mAP (%)
Random Embeddings	–	–	69.1	40.5
Learned Emb.	–	–	71.0	42.0
PPF (visual only)	–	–	71.3	42.2
Word2Vec	–	✓	71.5	42.3
BERT	bert-base	✓	72.1	42.7
CLIP Text Encoder	ViT-B/32	✓	**72.8**	**43.2**

**Table 8:** Computational efficiency comparison.

Method	Params (M)	GFLOPs	FPS	mAP_50_ (%)
Faster R-CNN [4]	41.5	134.4	18.2	58.3
DINO [36]	47.5	179.2	22.4	68.5
RT-DETR-L [15]	32.0	110.6	45.2	69.3
YOLOv9-C [13]	25.5	102.8	48.7	66.7
**SPFDet (Ours)**	38.7	156.3	35.6	**72.8**

## Data Availability

The HRSC2016 dataset is publicly available at https://www.kaggle.com/datasets/guofeng/hrsc2016 (accessed on 10 June 2026). The ShipRSImageNet dataset is publicly available at https://github.com/zzndream/ShipRSImageNet (accessed on 10 June 2026). The code, configuration files, prompt list, and evaluation scripts will be made available upon publication to facilitate reproducibility.

## Abbreviations

The following abbreviations are used in this manuscript:

SPFDet	CLIP Text-Prior-Guided Structure-Enhanced Detector
VLM	Vision-Language Model
CLIP	Contrastive Language-Image Pre-training
SPC	Semantic Prior Component
SETM	Structure-Enhanced Transformer Module
CCSM	Cross-level Channel Selection Module
MHCA	Multi-Head Cross-Attention
FCDF	Fused Category-Dominated Feature
CSC	Category-Semantic Consistency
FPN	Feature Pyramid Network
mAP	mean Average Precision
IoU	Intersection over Union
GIoU	Generalized Intersection over Union
